# Nutritional screening and extraglandular manifestations in Sjögren disease: a cross-sectional study using the controlling nutritional status score and prognostic nutritional index

**DOI:** 10.1186/s42358-026-00550-2

**Published:** 2026-06-09

**Authors:** Sibel Ösken, Duygu Şahin, Nihan Neval Uzun, Sibel Yılmaz Öner, Nesrin Şen, Mehmet Engin Tezcan

**Affiliations:** 1 Department of Rheumatology, Kartal Dr Lütfi Kırdar City Hospital, İstanbul, Türkiye; 2 Department of Rheumatology, İstanbul Erenköy Physical Therapy and Rehabilitation Hospital, İstanbul, Türkiye

**Keywords:** Controlling nutritional status score, Extraglandular manifestation, Sjögren disease, Prognostic nutritional index

## Abstract

**Background:**

Sjögren disease (SjD) is an autoimmune disorder marked by exocrine gland dysfunction and systemic extraglandular manifestations (EGM). While nutritional status plays a key role in chronic diseases, its association with EGM in SjD remains underexplored.

**Aims:**

This study aims to evaluate the nutritional status of patients with SjD using two validated tools the Controlling Nutritional Status (CONUT) score and the Prognostic Nutritional Index (PNI) and to examine their relationship with EGM.

**Methods:**

A cross-sectional analysis was conducted on 113 patients diagnosed with SjD, categorized into two groups: those with EGM (*n* = 26) and those without (*n* = 87). Nutritional status was assessed using serum albumin, total lymphocyte count, and total cholesterol for CONUT, and serum albumin with lymphocyte count for PNI. Clinical, serological, and laboratory data were collected, including the EULAR Sjögren’s Syndrome Disease Activity Index (ESSDAI).

**Results:**

The mean age of participants was 56.2 ± 10.9 years, with 94.7% being female. The EGM group exhibited a significantly higher prevalence of anti-Ro and anti-Ro-52 antibodies. Compared to the non-EGM group, the EGM group had significantly lower levels of albumin, white blood cells, neutrophils, lymphocytes, and PNI scores, and higher ESSDAI and CONUT scores. Multivariate logistic regression identified ESSDAI, CONUT and PNI scores were independently associated with the presence of EGM at diagnosis.

**Conclusion:**

This study underscores the importance of nutritional assessment in SjD, particularly in patients with systemic involvement. Malnutrition, as reflected by CONUT and PNI, is significantly associated with the presence of EGM. These findings highlight the need for routine nutritional screening in SjD management to support comprehensive care and improve patient outcomes.

## Key points

SjD patients with extraglandular manifestation had significantly lower PNI scores and higher CONUT scores.

CONUT and PNI scores were independently associated with the presence of EGM.

Routine nutritional assessment is recommended in SjD management to support comprehensive care and improve outcomes.

## Introduction

Sjögren disease (SjD) is a systemic autoimmune disorder primarily affecting the exocrine glands, resulting in xerostomia (dry mouth) and keratoconjunctivitis sicca (dry eyes). While these glandular features are hallmark symptoms, SjD can also involve multiple organ systems, including the skin, lungs, kidneys, nervous system, and joints. These extraglandular manifestations (EGM) are associated with increased disease burden, morbidity, and healthcare utilization [1]. SjD may manifest alone or alongside another underlying systemic autoimmune disease (associated) [2]. Its prevalence in the community ranges from 0.5% to 2.7% and is more prevalent in women (9:1 ratio) [3, 4]. Patients may experience various systemic manifestations, either initially or later, as outlined in the European League Against Rheumatism (EULAR) Sjögren’s Syndrome Disease Activity Index (ESSDAI), which is the primary parameter used to evaluate the disease’s activity. Severe extraglandular manifestations may occur in 20–40% of patients [5].

Malnutrition is an often-overlooked aspect of autoimmune diseases, particularly in SjD. Symptoms such as dry mouth, dysphagia, taste alteration, fatigue, and gastrointestinal involvement may impair dietary intake and nutrient absorption, leading to nutritional deficiencies. Moreover, systemic inflammation and metabolic disturbances may further exacerbate nutritional status [6].

The Controlling Nutritional Status Score (CONUT) and Prognostic Nutritional Index (PNI) have emerged as valuable tools for assessing nutritional status and predicting prognosis in various medical conditions. PNI, is a composite score reflecting an individual’s immunological nutritional status based on serum albumin and lymphocyte values. Another index used to assess immune nutritional status and detect undernutrition and poor nutritional status early is the CONUT score, which incorporates albumin concentration, lymphocyte count, and total cholesterol (TC) concentration. Both PNI and CONUT scores have been employed to identify malnutrition and long-term prognosis in patients with various malignancies [7]. Recently, these assessment indexes have been utilized to predict the activity and severity of several rheumatic diseases, such as systemic lupus erythematosus (SLE), antineutrophil cytoplasmic antibodies-associated vasculitis, rheumatoid arthritis (RA), Behçet’s disease (BD), and familial Mediterranean fever (FMF) [8–12]. In these studies, PNI and CONUT scores correlated with disease activity and severity. However, to our knowledge, no study has evaluated the relationship between PNI, CONUT scores, and SjD extra-glandular manifestations. This study aims to evaluate the nutritional status of patients with SjD using CONUT and PNI and to explore their relationship with EGM.

## Methods

### Patient selection and study design

We conducted a retrospective observational study by reviewing the medical records of 146 patients diagnosed with SjD who were receiving follow-up care at the Rheumatology Clinic of Kartal Lütfi Kırdar City Hospital between January 2009 and December 2022.

Inclusion criteria required a diagnosis of SjD according to the 2016 American College of Rheumatology (ACR)/European League Against Rheumatism (EULAR) classification criteria for SjD [13]. Exclusion criteria included a history of malignancy, chronic liver disease, the use of anticholinergic, antidepressant, antihistamine, diuretic, or neuroleptic medications, systemic vasculitis, sarcoidosis, amyloidosis, other autoimmune connective tissue diseases (e.g., systemic lupus erythematosus [SLE], rheumatoid arthritis [RA]), radiotherapy to the head or neck region, active infections (including hepatitis C virus and human immunodeficiency virus), graft-versus-host disease, IgG4-related disease, diabetes mellitus, end-stage renal disease, hypertension, and obesity.

**Fig. 1:**
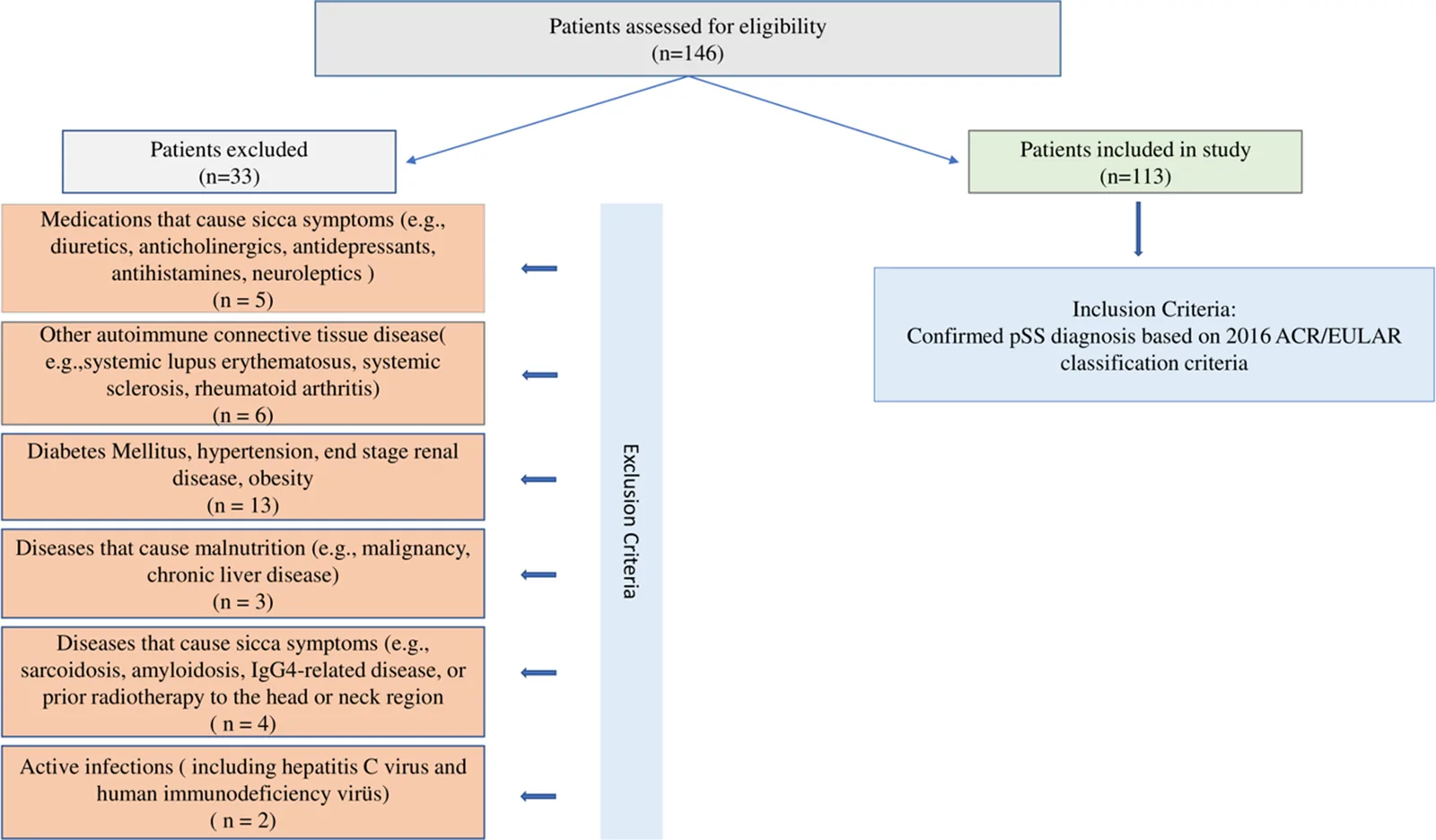
Study flow chart

After applying these exclusion criteria, 113 patients were eligible for the final analysis. A flowchart outlining the study population is presented in Fig. 1.

The study was approved by the Ethics Committee of Kartal Dr. Lütfi Kırdar City Hospital (Approval Date: 14.12.2022; Approval No: 2022/514/239/5) and conducted in accordance with the principles of the Declaration of Helsinki (1975, as revised).

### Data collection and definitions

Data were collected through a retrospective review of electronic medical records. Laboratory and demographic data were extracted from the hospital’s electronic health information system. The following laboratory parameters were recorded: white blood cell count (WBC), lymphocyte count, neutrophil count, platelet count, serum albumin, total cholesterol, C-reactive protein (CRP), erythrocyte sedimentation rate (ESR), and autoantibodies including antinuclear antibody (ANA), rheumatoid factor (RF), anti-Ro, anti-La, and anti-Ro-52. Focus scores from labial salivary gland biopsies were included when available.

The PNI was calculated using the following formula: PNI = (10 × serum albumin [g/dL]) + (0.005 × total lymphocyte count [/mm³]) [7].

The CONUT score was calculated based on three parameters: serum albumin, total cholesterol, and lymphocyte count. Each parameter was scored as follows: Albumin (g/dL): ≥3.5 = 0; 3.0–3.4 = 2; 2.5–2.9 = 4; <2.5 = 6, Total cholesterol (mg/dL): ≥180 = 0; 140–179 = 1; 100–139 = 2; <100 = 3, Lymphocyte count (/mm³): ≥1600 = 0; 1200–1599 = 1; 800–1199 = 2; <800 = 3 [12].

The total CONUT score was then classified into four nutritional risk categories: 0–1: Normal; 2–4: Mild; 5–8: Moderate; 9–12: Severe.

**Fig. 2:**
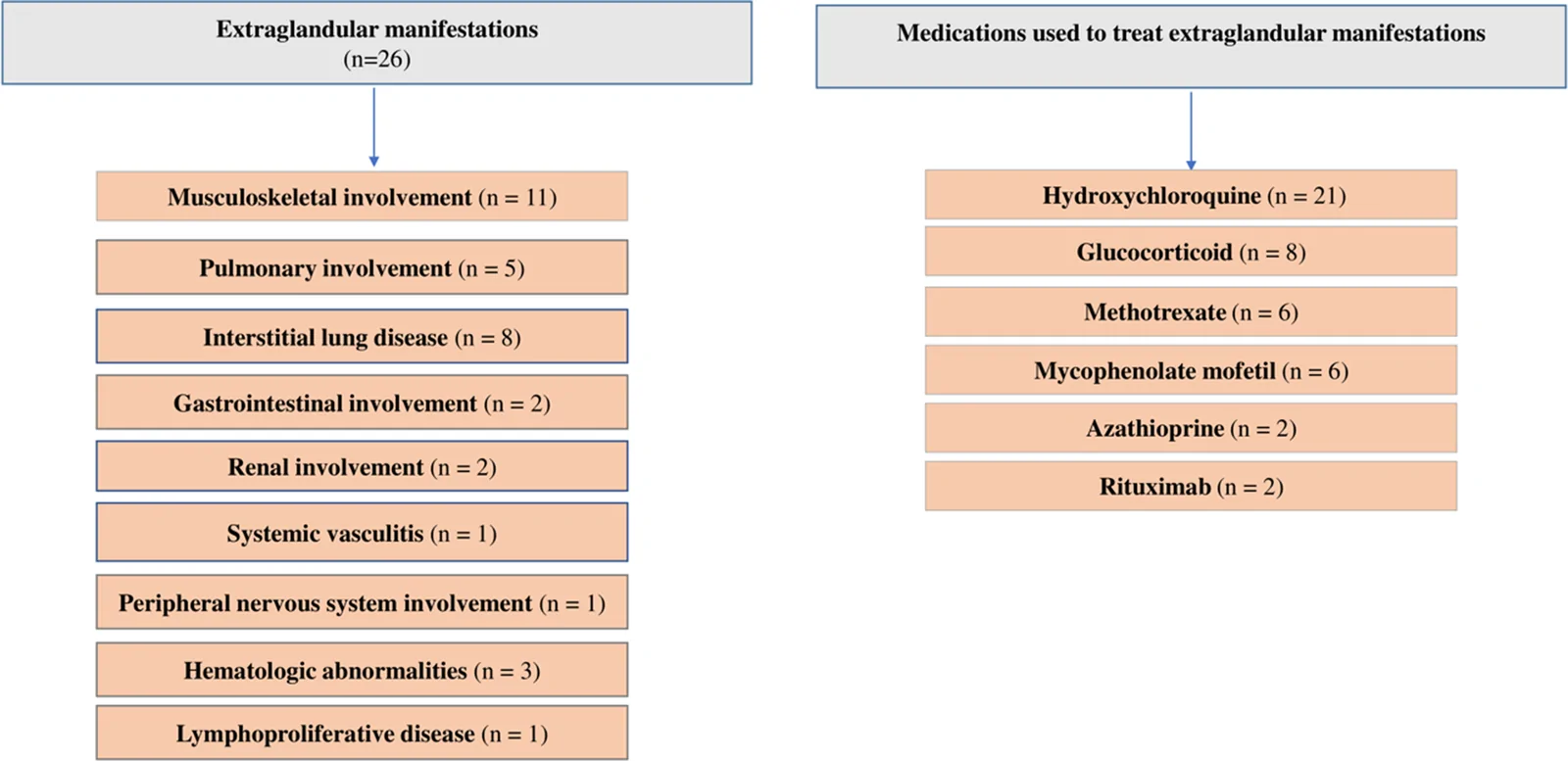
The types of extraglandular involvement and the corresponding medical treatment

Disease activity was evaluated using the EULAR Sjögren’s Syndrome Disease Activity Index (ESSDAI), which includes 12 organ-specific domains. Each domain is graded into three or four severity levels [13]. Patients were grouped into two categories according to the presence or absence of EGM. The types of extraglandular involvement and the corresponding medical treatments are shown in Fig. 2.

### Statistical analysis

All analyses were conducted using SPSS software, version 22.0 (IBM Corp., Armonk, NY).

A post hoc power analysis was conducted to evaluate the adequacy of the sample size. With a total of 113 participants, the study achieved an estimated statistical power of approximately 75% to detect a medium effect size (Cohen’s d = 0.5) at a two-sided significance level of 0.05 using a t-test. Although this is marginally below the conventional threshold of 80%, the power is considered acceptable for observational studies of this nature.

Data normality was assessed using the Kolmogorov–Smirnov test. Continuous variables were expressed as mean ± standard deviation (SD) or median (interquartile range), as appropriate. Categorical variables were reported as counts (percentages). Group comparisons were performed using the chi-square test, Fisher’s exact test, independent samples t-test, or the Mann–Whitney U test, as applicable. A p-value of < 0.05 was considered statistically significant. Multivariate logistic regression analysis was conducted to identify significant predictors of EGM. Receiver operating characteristic (ROC) curve analysis was used to evaluate the performance of CONUT and PNI levels in detecting the presence of EGM.

## Results

### Baseline characteristics

A total of 113 patients diagnosed with SjD were included in the study. Among them, 26 (23%) exhibited extraglandular manifestations (EGM), while 87 (77%) did not. The mean age of the entire cohort was 56.2 ± 10.9 years, with no significant difference between patients with and without EGM (57.9 ± 13.4 vs. 55.7 ± 10.1 years, respectively; *p* = 0.365). The majority of the cohort were female (94.7%), and gender distribution was similar between groups (*p* = 0.704).

Table 1 summarizes the baseline demographic characteristics, symptoms and laboratory findings (including autoantibodies) for both groups.

**Table 1:** Baseline characteristics of patients stratified by the presence of extraglandular manifestations

Characteristics	All patients (*n* = 113)	Extraglandular manifestation (+) (*n* = 26)	Extraglandular manifestation (-) (*n* = 87)	*P* value
Age, y, mean	56.2 ± 10.9	57.9 ± 13.4	55.7 ± 10.1	0.365
Gender, female	107 (94.7)	25 (96.2)	82 (94.3)	0.704
Current smoking	39 (34.5)	11 (42.3)	28 (32.2)	0.341
Weight (Kg)	70 (65-79.5)	69 (65.5–80)	70 (65–79)	0.948
Height (Cm)	161.1 ± 0.1	160.8 ± 0.8	161.2 ± 0.7	0.791
Body mass index	27.7 ± 4.4	27.6 ± 4.6	27.7 ± 4.4	0.912
Schirmer test (+)	95 (84.1)	21 (80.8)	74 (85.1)	0.600
Focus Score (biopsy)	1.48 ± 1.16	1.46 ± 1.39	1.49 ± 1.09	0.918
Symptoms – Organ Involvement				
Dry mouth	86 (76.1)	16 (61.5)	70 (80.5)	**0.047**
Dry eye	96 (85)	19 (73.1)	77 (88.5)	0.053
Autoantibodies				
ANA	78 (69.0)	21 (80.8)	57 (65.5)	0.140
Anti Ro	51 (45.1)	19 (73.1)	32 (36.8)	**0.001**
Anti Ro-52	42 (37.2)	15 (57.7)	27 (31)	**0.014**
Anti La	16 (14.2)	4 (15.4)	12 (13.8)	0.838
RF	25 (22.1)	7 (26.9)	18 (20.7)	0.502
Systemic Medications				
Pylocarpin	26 (23)	3 (11.5)	23 (26.4)	0.113
Cevimeline	8 (7)	2 (7.7)	6 (6.9)	0.890
Admission laboratory parameters				
Creatinine, mg/dl	0.7 (0.61–0.79)	0.69 (0.64–0.89)	0.7 (0.6–0.78)	0.143
Albumin, g/dl	4.2 (4.1–4.5)	4.1 (3.4–4.2)	4.3 (4.1–4.6)	**0.001**
Total cholesterol, mg/dl	192.55 ± 38.22	180.85 ± 38.99	196.05 ± 37.51	0.075
HDL-cholesterole, mg/dl	51.93 ± 17.03	53.67 ± 28.38	51.41 ± 11.92	0.554
LDL-cholesterole, mg/dl	119.27 ± 35.19	111.97 ± 32.79	121.45 ± 35.76	0.230
Hemoglobin, g/dl	12.66 ± 1.28	12.26 ± 1.47	12.78 ± 1.19	0.067
WBC, x10^9^/L	7.03 ± 2.21	5.82 ± 1.96	7.4 ± 2.16	**< 0.001**
Platelet, x10^9^/L	262.2 ± 66.3	259.9 ± 74.3	266.8 ± 64.2	0.645
Neutrophil, x10^9^/L	4.21 ± 1.66	3.55 ± 1.49	4.41 ± 1.67	**0.020**
Lymphocyte, x10^9^/L	2.07 ± 0.76	1.54 ± 0.72	2.23 ± 0.7	**< 0.001**
Monocyte, x10^9^/L	0.338 ± 0.320	0.401 ± 0.427	0.319 ± 0.282	0.256
Sedimentation, mm/h	23 (12–38)	30 (16.8–44.8)	22 (11–36)	0.084
CRP, mg/dl	5.6 (3.2–16.7)	12.3 (3.2–31.8)	5.2 (3.2–12.4)	0.061
ESSDAI	0.79 ± 1.99	2.3 ± 2.9	0.3 ± 1.3	**< 0.001**
CONUT Score	1.1 ± 1.7	2.6 ± 2.4	0.8 ± 1.1	**< 0.001**
PNI Score	53.3 ± 5.4	49.3 ± 6.2	54.5 ± 4.7	**< 0.001**

Abbreviations: CONUT, controlling nutritional status; CRP, C-reactive protein; ESSDAI, EULAR Sjögren disease disease activity index ; PNI, prognostic nutritional index; WBC, white blood cell

There were no statistically significant differences in current smoking status, body mass index (BMI), height, or weight between the two groups. The positivity rates for the Schirmer test and salivary gland biopsy focus scores were comparable between patients with and without EGM (85.1% vs. 80.8%, *p* = 0.600 and 1.49 ± 1.09 vs. 1.46 ± 1.39, *p* = 0.918, respectively).

However, dry mouth and dry eye symptoms were significantly less prevalent in patients with EGM compared to those without EGM (dry mouth: 61.5% vs. 80.5%, *p* = 0.047; dry eye: 73.1% vs. 88.5%, *p* = 0.053, marginal significance).

### Autoantibody profiles

The presence of anti-Ro antibodies was significantly higher in patients with EGM (73.1%) compared to those without (36.8%), (*p* = 0.001). Similarly, anti-Ro-52 positivity was more frequent in the EGM group (57.7% vs. 31%, *p* = 0.014). No significant differences were observed for ANA, anti-La, or RF positivity between the groups.

### Systemic treatments

There was a trend toward lower use of pilocarpine among patients with EGM compared to those without (11.5% vs. 26.4%), although this did not reach statistical significance (*p* = 0.113). The use of cevimeline was similar in both groups (*p* = 0.890).

### Laboratory findings

Patients with EGM exhibited significantly lower albumin levels compared to those without EGM (4.1 [3.4–4.2] vs. 4.3 [4.1–4.6] g/dL, *p* = 0.001). Additionally, WBC counts (5.82 ± 1.96 vs. 7.4 ± 2.16 × 10⁹/L, *p* < 0.001), neutrophils (3.55 ± 1.49 vs. 4.41 ± 1.67 × 10⁹/L, *p* = 0.020), and lymphocytes (1.54 ± 0.72 vs. 2.23 ± 0.70 × 10⁹/L, *p* < 0.001) were significantly lower in patients with EGM.

Although total cholesterol, LDL, and hemoglobin levels showed numerically lower trends in the EGM group, these did not reach statistical significance. No significant differences were found in creatinine, platelet, HDL cholesterol, or monocyte counts.

### Disease activity and nutritional indices

Disease activity, as assessed by the ESSDAI, was significantly higher in the EGM group (2.3 ± 2.9 vs. 0.3 ± 1.3, *p* < 0.001). Similarly, patients with EGM had significantly higher CONUT scores (2.6 ± 2.4 vs. 0.8 ± 1.1, *p* < 0.001) and lower PNI scores (49.3 ± 6.2 vs. 54.5 ± 4.7, *p* < 0.001).

### Predictors of extraglandular manifestations

Table 2 presents the results of the binary logistic regression analysis conducted to identify factors predicting extraglandular involvement in the study population.

**Table 2:** Univariate and multivariate analyses for predicting extraglandular manifestations in Sjögren disease

Univariate analysis	*P* value	OR (95% CI)	Multivariate analysis	*P* value	OR (95% CI)
Age	0.362	1.01 ( 0.97–1.06)			
Gender (Female)	0.706	1.52 (0.17–13.66)			
Current smoking	0.343	1.54 (0.62–3.79)			
Disease duration	0.754	0.99 (0.98–1.01)			
Anti Ro	**0.002**	4.66 (1.76–12.3)			
Anti Ro-52	**0.016**	1.52 (0.65–3.52)			
*WBC, x10^9^/L	**0.003**	1 (0.99–1)			
*Neutrophil, x10^9^/L	**0.024**	1 (0.99–1)			
*Lymphocyte, x10^9^/L	**< 0.001**	0.998 (0.998–0.999)			
ESSDAI	**< 0.001**	1.61 (1.24–2.09)	ESSDAI	**< 0.001**	1.88 (1.38–2.55)
CONUT Score	**< 0.001**	1.83 (1.37–2.46)	CONUT Score	**0.005**	1.84 (1.20–2.82)
PNI Score	**< 0.001**	0.80 (0.72–0.90)	PNI Score	**0.041**	0.84 (0.72–0.99)

Abbreviations: *CI*, confidence interval; *CONUT*, controlling nutritional status; *ESSDAI*, EULAR Sjögren’s syndrome disease activity index ; *OR*, Odds ratio; *PNI*, prognostic nutritional index; *WBC*, white blood cell

* The data on white blood cell (WBC), neutrophil, and lymphocyte counts, which overlap with CONUT and PNI scores, were not included in the stepwise backward logistic regression analysis

### Univariate analysis

In univariate logistic regression, several variables were significantly associated with the presence of EGM. Anti-Ro positivity was a strong predictor (OR: 4.66; 95% CI: 1.76–12.3, *p* = 0.002). Disease activity (ESSDAI) also showed a significant association (OR: 1.61; 95% CI: 1.24–2.09, *p* < 0.001), as did the CONUT score (OR: 1.83; 95% CI: 1.37–2.46, *p* < 0.001) and PNI (OR: 0.80; 95% CI: 0.72–0.90, *p* < 0.001). WBC, neutrophil, and lymphocyte counts were also significant predictors in univariate analysis; however, they were not included in the multivariate model due to overlap with CONUT and PNI scores.

### Multivariate analysis

In the multivariate logistic regression model, three independent predictors of EGM were identified: ESSDAI (OR: 1.88; 95% CI: 1.38–2.55, *p* < 0.001), CONUT score (OR: 1.84; 95% CI: 1.20–2.82, *p* = 0.005), PNI score (OR: 0.84; 95% CI: 0.72–0.99, *p* = 0.041) (Table 2).

**Fig. 3:**
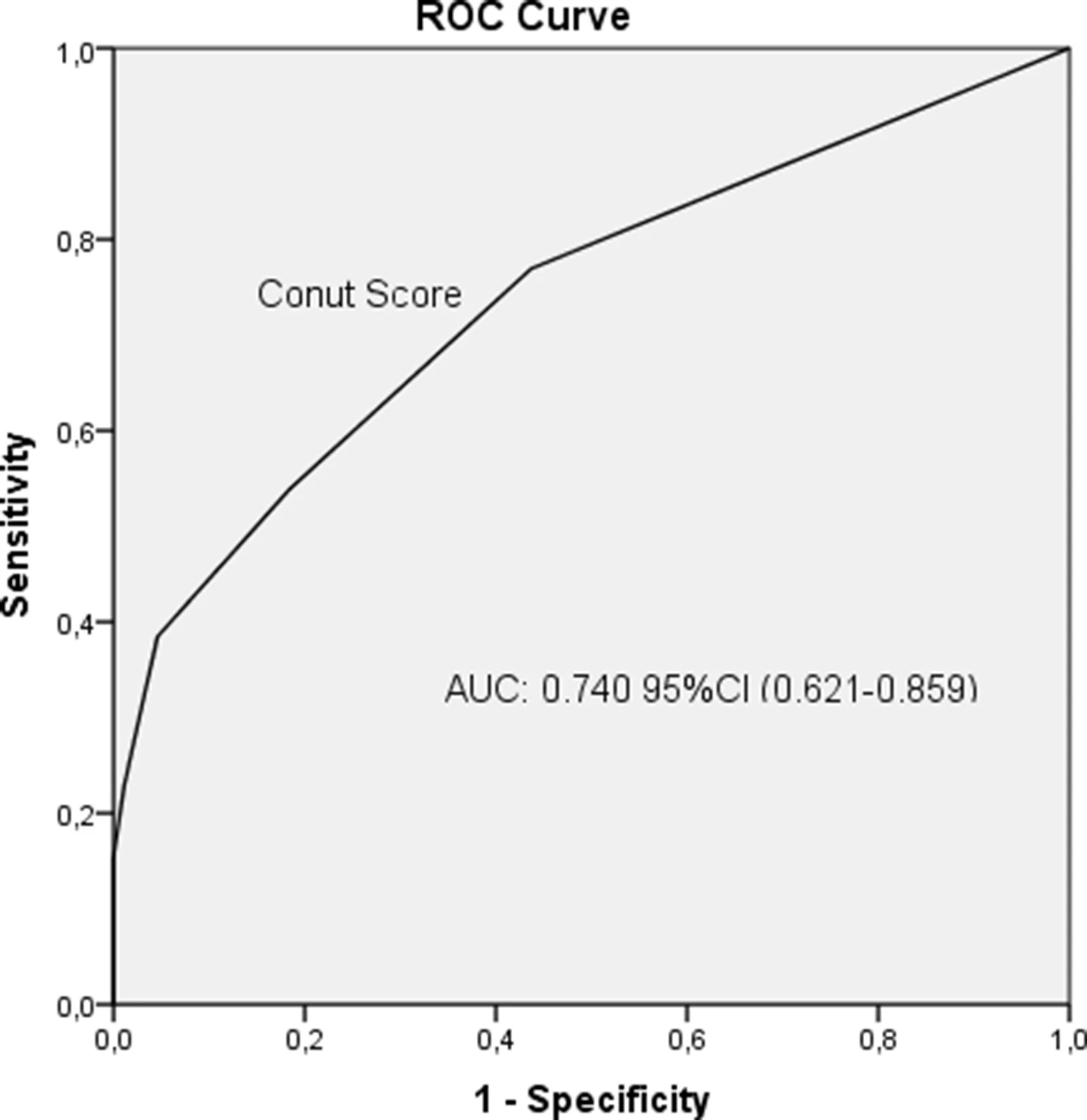
ROC curve analysis showing that a CONUT score > 3.5 had 95% specificity and 62% sensitivity for predicting EGM (AUC: 0.740, 95% CI: 0.621–0.859) AUC = area under the curve; CI = confidence interval; CONUT = controlling nutritional status; ROC = receiver operating characteristic

**Fig. 4:**
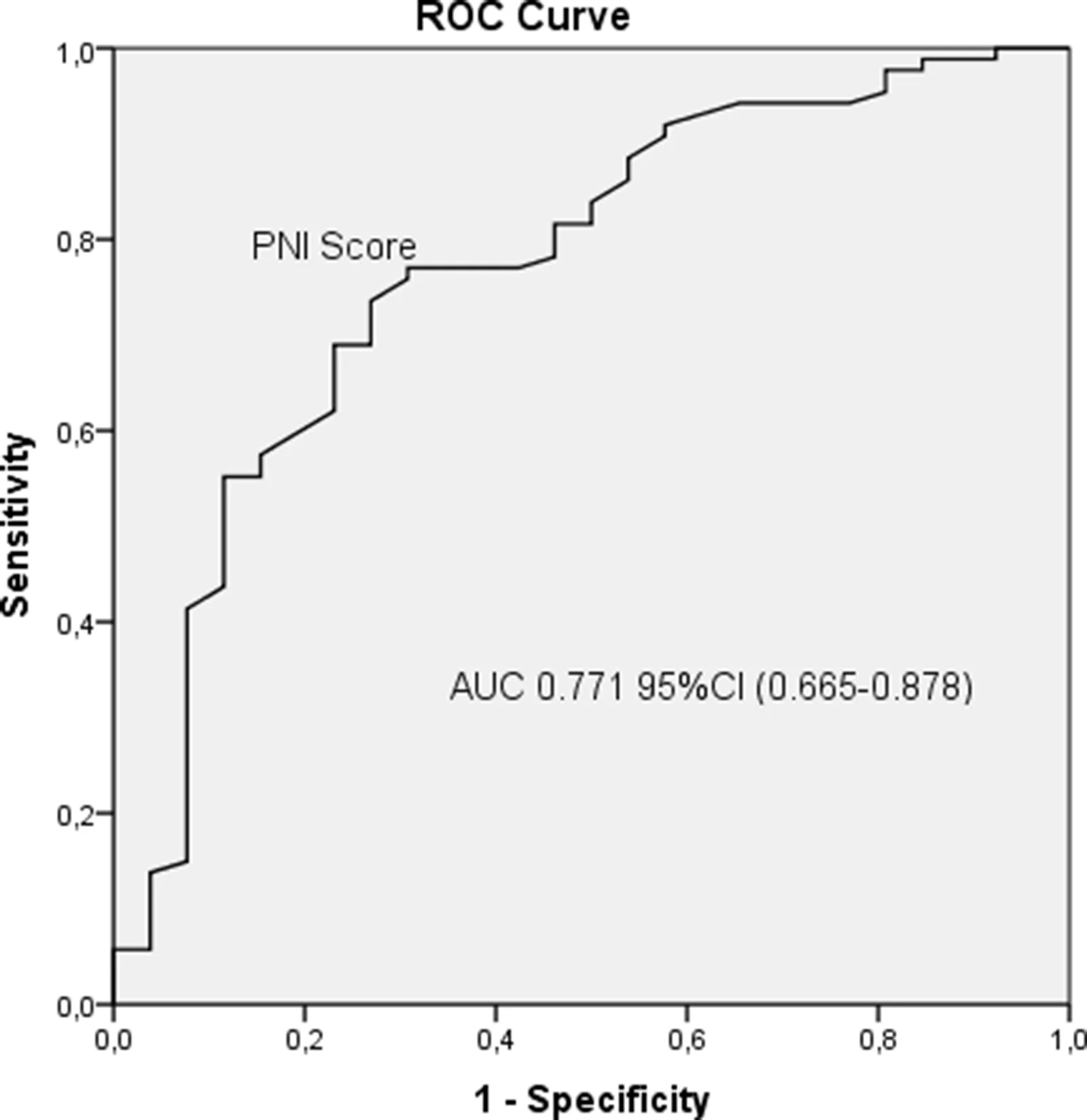
ROC curve analysis demonstrating that a PNI score > 51.625 had 74% specificity and 73% sensitivity for predicting EGM (AUC: 0.771, 95% CI: 0.665–0.878). AUC = area under the curve; CI = confidence interval; PNI = prognostic nutritional index; ROC = receiver operating characteristic

To evaluate the discriminative ability of CONUT and PNI scores for identifying the presence of EGM at diagnosis, receiver operating characteristic (ROC) curve analysis was performed. A CONUT score > 3.5 demonstrated 95% specificity and 62% sensitivity (area under the curve [AUC]: 0.740; 95% CI: 0.621–0.859). A PNI score > 51.625 yielded 74% specificity and 73% sensitivity (AUC: 0.771; 95% CI: 0.665–0.878) for discriminating EGM at diagnosis (Figs. 3 and 4).

## Discussion

To the best of our knowledge, this is the first study to investigate the relationship between nutritional status that assessed via the CONUT score and the PNI and EGM in patients with SjD. Our findings show a cross-sectional association indicating that patients with EGM exhibit significantly poorer nutritional status, reflected by elevated CONUT and reduced PNI scores, compared to those without EGM. Both nutritional indices were independently associated with the presence of EGM, alongside the ESSDAI, underscoring the potential clinical utility of routine nutritional assessment in SjD.

In this context, CONUT and PNI offer practical, objective, and cost-effective means of assessing nutritional and immune status. Recent evidence supports the relationship between nutritional indices and disease activity in multiple rheumatologic conditions. For instance, Rodríguez et al. reported that PNI and the Nutritional Risk Index (NRI) were significantly lower in patients with active SLE [8]. Ataş et al. identified a similar association in Behçet’s disease [9], while studies in familial Mediterranean fever (FMF) linked both PNI and CONUT scores to disease activity and amyloidosis risk [10].

These indices may provide indirect insight into immune dysregulation and systemic disease severity. Patients with more severe disease often exhibit poorer nutritional status, reinforcing the importance of integrating nutritional screening into standard clinical practice.

Malnutrition in SjD is a multifactorial condition. Common disease-related factors including xerostomia, dysgeusia, dysphagia, and gastrointestinal dysmotility can severely impair dietary intake. However, objective salivary flow measurements and cumulative oral damage indices such as the Sjögren’s Syndrome Damage Index (SSDDI) were not available in this retrospective study. Therefore, the relative contribution of glandular dysfunction–related nutritional impairment could not be fully delineated. Notably, despite comparable objective glandular involvement, patients with extraglandular manifestations reported fewer subjective sicca symptoms, which may reflect a systemic-dominant disease phenotype in which extraglandular involvement and inflammatory burden overshadow glandular symptom perception. This observation suggests that systemic inflammation rather than salivary dysfunction alone may play a predominant role in nutritional deterioration in this subgroup, and further highlights the heterogeneity of clinical presentation in Sjögren’s disease.

Simultaneously, chronic systemic inflammation may accelerate catabolism and impair nutrient absorption and metabolism [14]. These mechanisms contribute to hypoalbuminemia and lymphopenia which are the key components of both the CONUT and PNI scores. In addition, reduced total cholesterol levels, another component of the CONUT score may reflect inflammation-related alterations in lipid metabolism and increased metabolic consumption rather than insufficient dietary intake alone, particularly in the context of chronic autoimmune disease.

In our cohort, patients with EGM showed significantly reduced levels of albumin, white blood cells (WBC), neutrophils, and lymphocytes, consistent with systemic inflammation and potential nutritional depletion. Immunosuppressive and immunomodulatory therapies, which are more commonly prescribed in patients with extraglandular manifestations, may contribute to lymphopenia and thereby influence nutritional indices incorporating lymphocyte counts. Although treatment-related effects cannot be fully excluded, the persistence of CONUT and PNI as independently associated factors after adjustment for ESSDAI suggests that these indices reflect broader immunonutritional consequences of systemic inflammation rather than medication effects alone. Nevertheless, because detailed data on the type, duration, and cumulative exposure to immunosuppressive and immunomodulatory therapies were not available, residual confounding related to treatment exposure cannot be fully excluded. This supports the concept of malnutrition-inflammation complex syndrome (MICS), which in the context of Sjögren’s disease is best interpreted as a downstream consequence of sustained systemic inflammation rather than a primary driver of extraglandular manifestations [15]. Although nutritional impairment and systemic inflammation may coexist and potentially amplify each other, the present cross-sectional design does not allow determination of temporal directionality, and any bidirectional relationship should be considered hypothesis-generating. Future prospective studies incorporating nutritional assessment tools less dependent on lymphocyte counts may help to further clarify this relationship.

Based on our findings, we propose a conceptual model in which sustained systemic inflammation in Sjögren’s disease contributes to metabolic and immunological alterations, leading to measurable deterioration in nutritional indices such as CONUT and PNI. These changes may coexist with extraglandular manifestations and reflect overall disease burden rather than acting as independent pathogenic drivers. This framework is intended to be hypothesis-generating and does not imply causality.

ROC analysis demonstrated that both nutritional indices have moderate discriminative ability for identifying the presence of extraglandular manifestations at the time of diagnosis. A CONUT score > 3.5 yielded excellent specificity (95%), supporting its utility in distinguishing patients with and without EGM in a cross-sectional setting rather than predicting future disease development. Similarly, a PNI cut-off ≤ 51.625 showed balanced sensitivity and specificity (~ 73–74%), making it a practical screening tool for identifying EGM at diagnosis. Thus, the discriminatory performance of CONUT in this cohort likely reflects a composite of immunological, inflammatory, and metabolic disturbances present at the time of assessment rather than isolated nutritional deficiency or prospective prognostic value.

Importantly, our findings suggest that CONUT and PNI are not merely nutritional markers but may also reflect systemic immune dysregulation in SjD. In the present study, immune involvement was assessed indirectly through routinely measured laboratory parameters, including lymphocyte counts and serum albumin levels. Prior studies have reported associations between lymphocyte abnormalities, hypoalbuminemia, and increased systemic inflammatory burden; however, specific immune mechanisms such as immune complex deposition, chronic antigen stimulation, or functional immune impairment were not directly evaluated in this cohort. Therefore, the observed alterations in CONUT and PNI should be interpreted as markers of immune and inflammatory dysregulation rather than evidence of specific pathogenic immune mechanisms contributing to EGM. Any potential bidirectional or amplifying interaction between nutritional impairment and systemic involvement remains hypothesis-generating and requires confirmation in longitudinal studies incorporating functional immune assessments [16–18]. As such, worsening nutritional indices may mirror disease severity and systemic inflammatory burden.

The ESSDAI is a validated measure of systemic disease activity in SjD and was also independently associated with EGM in our study. It should be acknowledged that CONUT and PNI incorporate laboratory parameters that are influenced by systemic inflammation and may partially overlap conceptually with certain ESSDAI domains, particularly the hematologic domain. Accordingly, the independent association of CONUT and PNI after adjustment for ESSDAI should not be interpreted as evidence of distinct pathogenic pathways. Rather, these indices appear to capture complementary immunonutritional and metabolic consequences of systemic inflammation occurring within the same disease state.

The persistence of CONUT and PNI as significantly associated factors after adjustment for ESSDAI suggests that nutritional status captures complementary immunonutritional and metabolic consequences of systemic inflammation, within the same time frame, rather than representing a distinct pathogenic pathway or therapeutic target. Thus, CONUT and PNI may provide additional contextual information alongside ESSDAI, rather than serving as independent measures of disease activity. Nevertheless, residual confounding related to treatment exposure and disease severity cannot be entirely excluded. It should be acknowledged that the ESSDAI primarily reflects systemic disease activity, particularly extraglandular involvement, and includes a hematologic domain that may influence laboratory parameters incorporated into CONUT and PNI. Therefore, a certain degree of conceptual overlap is unavoidable. However, the persistence of CONUT and PNI as independently associated factors after adjustment for ESSDAI suggests that these indices may reflect complementary immunonutritional and metabolic consequences of systemic inflammation rather than redundant measures of disease activity.

EGM in SjD are associated with increased disease burden, poorer quality of life, and higher morbidity and mortality. Their management often requires a multidisciplinary approach and presents diagnostic and therapeutic challenges. Therefore, early recognition and appropriate intervention are critical to optimizing outcomes. Nutritional deterioration in SjD may stem from reduced oral intake, gastrointestinal dysfunction, and increased metabolic demands. While comprehensive nutritional assessments including dietary intake, anthropometry, biochemical markers, and subjective global assessment are ideal, there remains a need for simple, efficient tools that can predict adverse outcomes and guide clinical management.

### Limitations

This study has several strengths, including the comprehensive exclusion of confounding comorbidities (e.g., diabetes, obesity, and malignancy), robust statistical analysis, and standardized assessment of nutritional and disease activity scores. At the same time, this study has several limitations. First, single-center design limits generalizability; due to the retrospective cross-sectional design, causal relationships and temporal associations could not be established; and potential confounders affecting CONUT/PNI were not fully controlled, and both ESSDAI and laboratory data were obtained through the review of medical records. Second, temporal changes could not be assessed, as all measurements were taken only at the time of diagnosis. Third, variables such as patients’ dietary habits, physical activity levels, and use of nutritional supplements were not evaluated, which may have influenced the results. Additionally, objective measures of salivary gland function (e.g., salivary flow rates) and cumulative oral damage indices such as the Sjögren’s Syndrome Damage Index (SSDDI) were not available, limiting our ability to assess the contribution of glandular dysfunction to nutritional status. Moreover, detailed information on immunosuppressive and immunomodulatory treatment exposure was not available, limiting our ability to fully assess their potential impact on lymphocyte-based nutritional indices. Prospective, longitudinal studies with serial assessments are needed to evaluate the effects of treatment and other clinical features of SjD on PNI and CONUT scores.

## Conclusion

This study highlights the often-overlooked relationship between nutritional status and systemic involvement in SjD. The results demonstrate that patients with EGM are at greater risk of malnutrition, as evidenced by higher CONUT scores and lower PNI values. Both CONUT and PNI are practical, low-cost tools derived from standard laboratory tests, making them feasible for routine use in rheumatology clinics. Their correlation with disease severity and systemic involvement supports their utility in risk stratification and treatment planning.

## Data Availability

The datasets generated and/or analyzed during the current study are available from the corresponding author upon reasonable request.
